# Cytosolic Extract Induces Tir Translocation and Pedestals in EPEC-Infected Red Blood Cells

**DOI:** 10.1371/journal.ppat.0040004

**Published:** 2008-01-18

**Authors:** Alyson I Swimm, Daniel Kalman

**Affiliations:** Department of Pathology and Laboratory Medicine, Emory University, Atlanta, Georgia, United States of America; Yale University School of Medicine, United States of America

## Abstract

Enteropathogenic *Escherichia coli* (EPEC) are deadly contaminants in water and food, and induce protrusion of actin-filled membranous pedestals beneath themselves upon attachment to intestinal epithelia. Pedestal formation requires clustering of Tir and subsequent recruitment of cellular tyrosine kinases including Abl, Arg, and Etk as well as signaling molecules Nck, N-WASP, and Arp2/3 complex. We have developed a cytosolic extract-based cellular system that recapitulates actin pedestal formation in permeabilized red blood cells (RBC) infected with EPEC. RBC support attachment of EPEC and translocation of virulence factors, but not pedestal formation. We show here that extract induces a rapid Ca^++^-dependent release of Tir from the EPEC Type III secretion system, and that cytoplasmic factor(s) present in the extract facilitate translocation of Tir into the RBC plasma membrane. We show that Abl and related kinases in the extract phosphorylate Tir and that actin polymerization can be reconstituted in infected RBC following addition of cytosolic extract. Reconstitution requires the bacterial virulence factors Tir and intimin, and phosphorylation of Tir on tyrosine residue 474 results in the recruitment of Nck, N-WASP, and Arp2/3 complex beneath attached bacteria at sites of actin polymerization. Together these data describe a biochemical system for dissection of host components that mediate Type III secretion and the mechanisms by which complexes of proteins are recruited to discrete sites within the plasma membrane to initiate localized actin polymerization and morphological changes.

## Author Summary

*Enteropathogenic Escherichia coli* (EPEC) is a diarrheagenic enteric pathogen that attaches to host cells and forms actin-filled membranous protrusions called pedestals. Pedestal formation is initiated when the EPEC virulence factor, Tir, is translocated into the host cell via bacterial Type III secretion (T3S) and inserted into the plasma membrane, initiating a signaling cascade that results in actin polymerization beneath attached bacteria. We have developed a cytoplasmic extract-based system in permeabilized cells to study these early events in EPEC pathogenesis, many of which are not easily studied in intact cells. We have taken advantage of the observation that EPEC fail to form pedestals on red blood cells (RBC). We report that low calcium triggers T3S of Tir into RBC, indicating how the T3S apparatus may sense entry into the host cytoplasm. Additionally, insertion of Tir into the host membrane depends upon host cytoplasmic components, a requirement not previously recognized or accessible to experimental manipulation in intact cells. Finally, cytoplasmic extract reconstitutes actin polymerization beneath attached bacteria using signaling molecules required in intact cells. We are currently purifying the components that mediate these processes. Together, these experiments show how functional biochemistry approaches can reveal novel roles for cytoplasmic factors in host–pathogen interactions.

## Introduction

Pathogenic E. coli strains, including enteropathogenic *E. coli* (EPEC) and enterohemorrhagic *E. coli* (EHEC), contaminate food and water supplies and colonize the gastrointestinal tract, causing inflammation, diarrhea and death, particularly among children in developing nations [1]. A distinguishing feature of EPEC and EHEC is the production of attaching and effacing (A/E) lesions in the intestine [2], which are characterized by retraction of intestinal microvilli and formation of actin-filled protrusions or “pedestals” beneath attached bacteria [3].

Pedestal formation is initiated through secretion of bacterial virulence factors, including the translocated intimin receptor (Tir), into the host cell via a Type III secretion apparatus [4]. Tir is incorporated into the host cell plasma membrane where it binds intimin, a bacterial outer membrane protein [5]. Interaction with intimin clusters Tir, a process that is required for localized cytoskeletal changes [4,6,7], thereby initiating a signaling cascade involving recruitment of tyrosine kinases which phosphorylate Tir [4,8,9], and likely other bacterial or host proteins [7].

Our previous work has shown that Abl, Arg and Etk are recruited and activated in EPEC pedestals, and can directly or indirectly phosphorylate Tir or other pedestal proteins [10,11]. Work of Phillips et al. [12], has suggested that Fyn also participates in pedestals induced using a dual bacterial infection system. However, because pedestals induced by EPEC form on cells lacking Src, Fyn and Yes [11,13], this kinase alone does not appear to be necessary. Indeed, tyrosine kinases appear to act in a redundant fashion. Thus, any one kinase is capable of supporting actin polymerization in the absence of the other [10,11]. Stable kinase recruitment via polyproline and phosphotyrosine residues within Tir, and SH3 and SH2 domains within the kinases, is required for recruitment of downstream effectors and for pedestal formation [10]. Such effectors include the host signaling molecules Nck [14], N-WASP, and the Arp2/3 complex [15], which catalyze actin polymerization beneath attached bacteria.

While much is now known about the mechanisms by which kinases and other signaling factors are recruited and activated once Tir is clustered, less is known about whether host cytoplasmic components contribute to the insertion, clustering, or anchoring of Tir in the plasma membrane, or whether kinases phosphorylate targets other than Tir to induce subsequent cytoskeletal changes. Such questions have proven difficult to address, in part because of a lack of genetic and biochemical systems with which to study pedestal formation.

We considered the possibility that infection of red blood cells (RBC) with EPEC might be a useful means to study recruitment and activation of signaling molecules at the plasma membrane. EPEC initially adheres to RBC in vitro by EspA filaments and subsequently induces the formation of pores in the RBC plasma membrane, resulting in hemolysis [16–18]. EPEC then translocates Tir into the RBC membrane and initiates Tir/intimin interaction across the membrane, as seen in other cell types. However, RBC do not support tyrosine phosphorylation of Tir and, therefore, actin pedestals do not form beneath attached bacterium [19]. It remains possible that no suitable kinase exists in this cell type, or that the phosphorylation site on Tir is masked, or that Tir is not appropriately clustered or inserted into the RBC plasma membrane to facilitate kinase recruitment. We reasoned that it might be possible to extend the findings of Shaw and colleagues and explore the translocation of Tir and the recruitment of signaling molecules in RBC infected with EPEC by reconstituting pedestal formation with cytosolic extract.

Permeabilized cells and cytosolic extract systems have been used to explore a multitude of problems in cell biology and microbial pathogenesis. For example, cellular extracts support actin based vesicle motility [20] as well as actin “tail” formation and motility by such cytoplasmic pathogens as Listeria monocytogenes [21]. Additionally, extract-based systems have proven critical in purifying components of the actin polymerization machinery, such as the Arp2/3 complex [22–25], and permeabilized cell systems have been used to determine sites of newly polymerized actin [26]. Finally, cell-free extracts have been used in conjunction with purified components to reconstitute actin motility of L. monocytogenes and *Shigella flexneri* [27]. Notably, a phosphorylated 12-mer containing sequences surrounding Y474 within Tir is sufficient to induce actin polymerization in cytoplasmic extracts [6].

Using cytosolic extracts in conjunction with permeabilized RBC infected with EPEC, we show here that a decrease in calcium mediates rapid release of Tir from the EPEC Type III secretion system, and that cytoplasmic components facilitate translocation of the released Tir into the host plasma membrane. We also show that addition of extract induces tyrosine phosphorylation of Tir and subsequent actin polymerization beneath attached EPEC and that these processes require the same host signaling mechanisms used to form pedestals on intact cells. These data illustrate the utility of functional biochemistry approaches in understanding host-pathogen interactions.

## Results

### Reconstitution of Actin Pedestals in Human RBC Infected with EPEC

**Figure 1:**
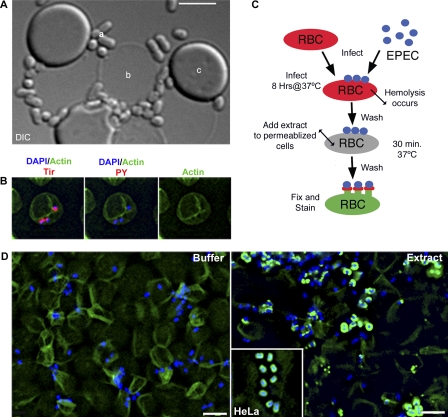
Exposure of RBC to Cytosolic Extract Induces Actin Polymerization beneath Attached EPEC (A) DIC image of EPEC (a) adhering to RBC that had undergone hemolysis (b) or remained intact (c) 6 h after infection. (B) Images of RBC infected with EPEC and stained with DAPI to visualize bacteria (blue), Alexa-488-phalloidin to visualize actin (green), anti-Tir antibody (left panel), and anti-phosphotyrosine (PY) 4G10 antibody (middle panel). Note the lack of PY and actin accumulation beneath the bacteria. (C) Schematic of method used to induce pedestal formation in RBC. (D) Images of EPEC infected RBC after exposure to either buffer or extract for 20 min. Inset in D depicts actin pedestals formed on a HeLa cell infected with EPEC. Cells are stained with DAPI to identify EPEC (blue) and Alexa-488 phalloidin to visualize actin (green). Scale bar in all images represents 5 μm.

In initial experiments, human RBC were plated and infected with EPEC from an overnight culture, as previously described [16,19]. In accordance with previous reports [16,17], the majority of RBC underwent hemolysis during infection and EPEC remained adhered to the lysed RBC membranes (Figure 1A). Upon visualization of Tir, phosphotyrosine and actin by indirect immunofluorescence microscopy, Tir was present beneath a small fraction of attached EPEC, but no associated phosphotyrosine or actin staining was evident, in agreement with previous reports [19] (Figure 1B), even with prolonged infections (up to 16 h, the longest time tested). By contrast, infection of HeLa cells with EPEC for as little as four hours induced formation of characteristic actin “pedestals”, visualized as intense actin staining apposed to the bacteria (Figure 1D; inset).

To reconstitute actin pedestals, monolayers of infected RBC were incubated with a solution containing Triton X-100, protease inhibitors, an ATP-regeneration system, and cytosolic extract prepared from either homogenized brain of C57BL/6 mice, or frozen porcine brain (together called “extract”; see Material and Methods) for 20 min at 37 °C (Figure 1C). Control samples were identically treated except that the extract in the reaction mixture was replaced with buffer. As seen in Figure 1D, intense actin staining (green) was evident beneath many of the attached bacteria (blue) following addition of extract for 20 min. By contrast, no actin staining was evident after treatment with buffer, although faint actin staining of the RBC membranes could be seen at this exposure in both conditions.

The actin beneath attached EPEC in RBC never appeared elongated, a common feature of pedestals seen on 3T3 cells. Rather, the actin appeared in “cups” surrounding attached bacteria. Such cups were reminiscent of shorter pedestals seen in some cell types (e.g., HeLa; Figure 1D; inset). Actin polymerization induced by extract was transient and was evident only between 10 and 30 min following the addition of extract to infected RBC. This transiency was apparent even when the extract was repeatedly replenished, suggesting that proteolysis, inactivation, or “washout” of components critical for sustaining the process occurred at longer incubation times.

### Extract-Induced Actin Polymerization in RBC Requires the Same Bacterial and Cellular Components as Pedestals on Intact Cells

**Figure 2:**
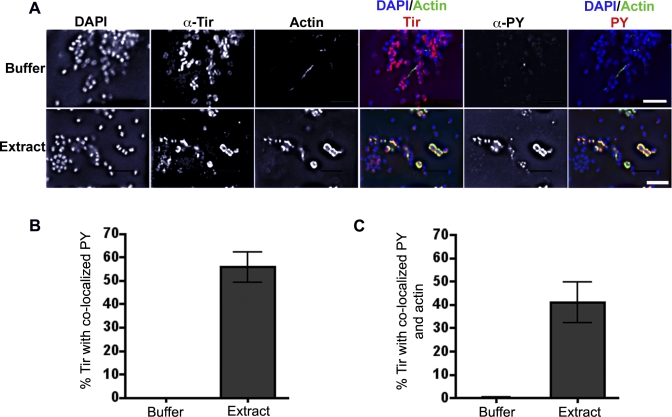
Actin Colocalizes with Tir and Phosphotyrosine (PY) Staining in RBC Infected with EPEC and Exposed to Extract (A) Images of RBC infected with EPEC and exposed to either buffer or extract. Cells are labeled with DAPI to identify EPEC, Alexa-488 phalloidin to visualize actin, and either anti-Tir antibody or anti-PY 4G10 antibody. Note that tyrosine phosphorylation and actin polymerization in extract-treated RBC is colocalized with Tir. (B) Quantitative analysis of colocalization of Tir and PY staining beneath attached EPEC in RBC exposed to either buffer or extract. (C) Quantitative analysis of colocalization of Tir, PY, and actin staining beneath attached EPEC in RBC exposed to either buffer or extract. Scale bars in all images represent 5 μm.

We next set out to determine whether intense actin staining seen beneath EPEC in RBC exposed to extract was characteristic of pedestals formed by EPEC on other cell types. As in HeLa or 3T3 cells infected with EPEC, Tir and phosphotyrosine colocalized with intense actin staining directly beneath attached bacteria on RBC treated with extract (Figure 2A). Quantitation of colocalization of Tir with phosphotyrosine and actin staining (see Materials and Methods) indicated that of those bacteria attached to RBC and expressing Tir, ∼60% were colocalized with phosphotyrosine (Figure 2B), and ∼45% with phosphotyrosine and intense actin staining (Figure 2C) following exposure to extract. Accordingly, the intense actin staining was only observed in conjunction with staining for bacteria, phosphotyrosine, and Tir. No colocalization of Tir with actin or phosphotyrosine was evident in RBC treated with buffer alone (Figure 2A). Thus, as in pedestals on intact cells, actin polymerization on extract treated RBC was colocalized with phosphotyrosine staining.

**Figure 3:**
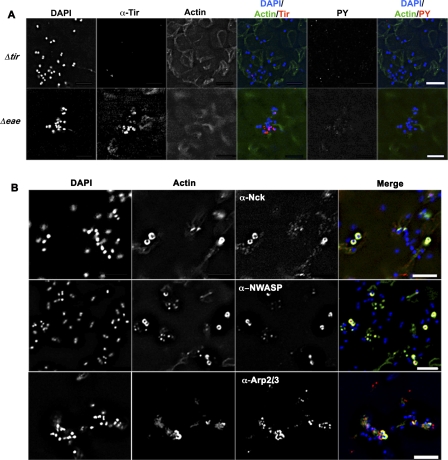
Actin Polymerization Induced by Extract in EPEC-Infected RBC Utilizes Both Bacterial and Host Factors (A) Images of RBC infected with EPEC mutants lacking Tir (EPEC*Δtir*) or intimin (EPEC*Δeae*) and treated with extract. Cells are labeled with DAPI to identify EPEC, Alexa-488 phalloidin to visualize actin, and either anti-Tir antibody or anti-PY 4G10 antibody. (B) Images of RBC infected with EPEC and treated with extract. Cells are labeled with DAPI to identify EPEC, Alexa-488-phalloidin to visualize actin, and either anti–Nck antibody, anti-WASP antibody, or anti-Arp 2/3 p21 antibody. Scale bars in all images represent 5 μm.

Interactions between intimin, located in the outer membrane of EPEC, and Tir, in the plasma membrane of the infected cell, induce Tir clustering, and are required for pedestal formation and for efficient phosphorylation of Tir [6]. To determine whether interactions between Tir and intimin were required for actin polymerization induced by extract in RBC, we tested EPEC strains deficient in either Tir (EPEC*Δtir*) or intimin (EPEC*Δeae*) for their ability to induce actin polymerization after treatment with extract. As seen in Figure 3A, both EPEC*Δeae* and EPEC*Δtir* failed to induce tyrosine phosphorylation or actin polymerization apposed to bacteria after exposure to extract. Together these data suggest that, as in other models of EPEC infection, interactions between Tir and intimin are critical for actin polymerization induced beneath EPEC in RBC exposed to extract.

Although Tir and intimin are critical bacterial components of EPEC signaling, the host signaling molecules Nck, N-WASP and the Arp2/3 complex are also required for pedestal formation by EPEC, and all of these proteins are recruited to pedestals directly beneath attached bacteria in HeLa and 3T3 cells [14,15]. We examined whether these proteins were also associated with EPEC and extract-induced actin polymerization on RBC. As seen in Figure 3B, Nck, N-WASP and the Arp2/3 complex were each evident colocalized with actin and EPEC in RBC exposed to extract. No staining was evident in cells treated with buffer alone (not shown), nor with bacteria that failed to induce actin polymerization in the presence of extract (Figure 3B). Nck, N-WASP, and Arp2/3 complex were present at low levels in RBC compared to extract by western analysis (data not shown), and likely washed out upon permeabilization. Thus we presume that the Nck, N-WASP and Arp2/3 complex evident beneath attached EPEC were derived from the extract. Together, these data indicate that the same signaling molecules that are required for pedestal formation on intact tissue culture cells are also recruited to sites of extract-induced actin polymerization on infected RBC.

### EPEC Tir Undergoes Modification and Phosphorylation on Y474 Upon Exposure to Extract

Following translocation into the host plasma membrane, Tir has been found to undergo tyrosine phosphorylation at Y474, an event required for EPEC pedestal formation. Recent data indicate that phosphorylation at Y454 may also catalyze actin polymerization, although less efficiently than Y474 [28]. Tir has also been shown to undergo phosphorylation at serine and threonine residues, which has been reported to facilitate pedestal elongation [29]. Serine and threonine, but not tyrosine modifications result in a sequential shift in the molecular mass of Tir on SDS-PAGE gels from ∼78 kDa (non-translocated Tir) to ∼82 kDa and ∼85 kDa [29] after translocation into the host membrane. A previous report indicated that in RBC infected with EPEC, Tir fails to undergo any host modifications upon translocation into the RBC membrane [19].

**Figure 4:**
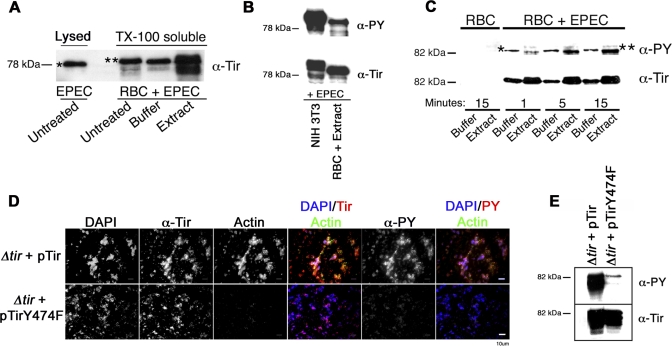
EPEC Tir Undergoes Modification in RBC Membranes and Is Rapidly Phosphorylated on Y474 upon Exposure to Extract (A) Western analysis using anti-Tir antibody of lysed EPEC (lane 1) or the TX-100 soluble fractions of RBC infected with EPEC and left untreated (lane 2), or treated with buffer or extract for 30 min (lanes 3 and 4). Note that the molecular weight of Tir increases from 78 kDa (*) (untranslocated Tir) to ∼82 kDa (**) upon infection of RBC, regardless of any post-infection treatment. Also note the increase in the amount of Tir in the TX-100 soluble fraction of infected RBC exposed to extract. (B) Western analysis using anti-Tir antibody and anti-PY 4G10 antibody of the TX-100 soluble fraction of 3T3 cells infected with EPEC (lane1), or RBC infected with EPEC and exposed to extract for 20 min (lane 2). Note that Tir from RBC infected with EPEC and exposed to extract is tyrosine phosphorylated, but does not undergo the same shift in molecular weight seen in infected 3T3 cells. (C) Western analysis using anti-Tir antibody and anti-PY 4G10 antibody in the TX-100 soluble fraction of either uninfected (lanes 1 and 2) or infected (lanes 3–8) RBC exposed to extract or buffer for 1, 5, or 15 min. Note that Tir is rapidly phosphorylated on the 82 kDa species after exposure to extract (**). A tyrosine phosphorylated protein of approximately the same molecular weight as Tir was evident following infection of RBC and exposure to buffer (*), but further analysis determined that this phosphoprotein was not Tir. (D) Images of RBC infected with EPEC*Δtir* mutants overexpressing either WT Tir (*Δtir* + pTir ) or Y474F Tir (*Δtir* + pTirY474F) and exposed to extract for 20 min. Cells are labeled with DAPI to identify EPEC, Alexa-488-phalloidin to visualize actin, and either anti-Tir antibody or anti-PY 4G10 antibody. Note the lack of actin polymerization and PY staining in RBC infected with EPEC*Δtir* + pTirY474F. Scale bars represent 10 μm. (E) Western analysis using anti-Tir antibody and anti-PY 4G10 antibody of the TX-100 soluble fraction of RBC infected with EPEC*Δtir* + pTir or EPEC*Δtir* + pTirY474F and exposed to extract. Note that no phosphorylation of Tir is evident in RBC infected with EPEC *Δtir* + pTirY474F.

We next determined whether Tir underwent any changes in molecular mass following infection of RBC and exposure to extract. To do this, RBC were infected with EPEC and exposed to buffer or extract, or left untreated, and the Triton X-100 (TX-100) soluble fraction was subjected to SDS-PAGE and western analysis with anti-Tir antibody to assess membrane-associated Tir. Lysates from overnight cultures of EPEC, or TX-100 soluble fractions from HeLa cells previously infected with EPEC were prepared as controls. In samples that were not treated with extract or buffer, but were washed and lysed immediately after infection, we noted a slight increase in the molecular mass of Tir upon infection of RBC compared to Tir in EPEC lysates from overnight cultures (Figure 4A, lane 1 and lane 2). The increase in molecular mass was small, from ∼78 kDa to ∼82 kDa (Figure 4A), but was consistently observed. When infected RBC were exposed to extract, an apparent increase in the amount of Tir present in the TX-100 soluble fraction was observed, but no additional increase in molecular mass was evident (Figure 4A, lane 4). Tir from infected RBC exposed to buffer alone also did not show any additional changes and ran at the same molecular mass as untreated, infected RBC (Figure 4A, lane 3). Notably, the change in molecular mass of Tir induced upon infection of RBC did not correspond to that observed in 3T3 cells infected with EPEC (Figure 4B, bottom panel; see also [29]). These results indicate that Tir undergoes partial host modification upon translocation into the RBC membrane and that this partially modified Tir is sufficient to support actin polymerization in the presence of extract.

We next determined whether Tir from infected RBC was tyrosine phosphorylated upon exposure to extract. RBC were infected with EPEC and exposed to extract for 20 min and the TX-100 soluble fraction was directly compared to that from infected 3T3 cells. As seen in Figure 4B, exposure of infected RBC to extract readily phosphorylated the 82 kDa molecular weight species (Figure 4B, lane 2). By contrast, an 85 kDa species was phosphorylated in infected 3T3 cells. No such phosphorylated band was present in the TX-100 soluble fraction from uninfected RBC or from RBC infected with EPECΔ*tir* and exposed to extract (Figure S1A), confirming that this band represents Tir. To determine the rate of phosphorylation of Tir following exposure to extract, RBC were infected and exposed to extract or buffer for 1, 5, or 15 min, and then subjected to SDS-PAGE and Western analysis. As seen in Figure 4C, upon exposure to extract, tyrosine phosphorylation of Tir was evident within one min and increased over time. Notably, a tyrosine phosphorylated protein of nearly the same molecular weight as Tir was also evident following infection of RBC, but only in the absence of extract (Figure 4C, lanes 3, 5 and 7). Although this phosphorylated protein appeared specific to RBC infected with EPEC and was not present in uninfected cells treated with either buffer or extract (Figure 4C, lanes 1 and 2), reprobing the blot with anti-Tir antibody clearly indicated that this band did not correspond to Tir. Heating the lysate to 90 °C prior to SDS-PAGE and removing the resulting precipitate, a procedure known to enrich for Tir in the supernatant [4], eliminated this band from the sample and this procedure was used in most of the subsequent experiments. Notably, as with actin polymerization, phosphorylation of Tir by extract was not sustained over time. Incubations with extract of greater than 30 min, even in the presence of phosphatase inhibitors, resulted in Tir dephosphorylation (Figure S1B), and mirrored the loss of actin polymerization at these times.

To determine whether the observed phosphorylation of Tir was functionally relevant, we tested the effects of an amino acid change from Y to F at position 474 (Tir-Y474F), a residue critical for pedestal formation in intact cells [8]. Tir-Y474F was expressed in an EPEC strain lacking Tir (EPEC*Δtir*) under control of its own promoter, and in conjunction with the Tir chaperone CesT [10]. RBC were infected with either EPEC*Δtir*+pTir or EPEC*Δtir*+pTir-Y474F and exposed to extract for 20 min. As seen in Figure 4D (or in an enlarged format in Figure S2), although Tir and Tir-Y474F were both colocalized beneath bacteria, actin polymerization and tyrosine phosphorylation were only evident in RBC infected with EPEC*Δtir* expressing wild-type Tir, but not Tir-Y474F (compare panels in columns 3 and 5 in Figure 4D). Western analysis confirmed that addition of extract induced phosphorylation of wild-type Tir in the TX-100 fraction of infected cells. By contrast, only residual phosphorylation was evident on Tir-Y474F (Figure 4E). The residual level observed may represent phosphorylation of Y454 [28], but was not associated with actin polymerization in the RBC system and was not characterized further. Together these data indicate that extract induces tyrosine phosphorylation of Tir on Y474 in EPEC infected RBC and that this phosphorylation is critical for actin polymerization in reconstituted RBC, just as it is in intact cells.

### PD-166326-Sensitive Tyrosine Kinases in the Extract Phosphorylate Tir and Are Required for Actin Polymerization

**Figure 5:**
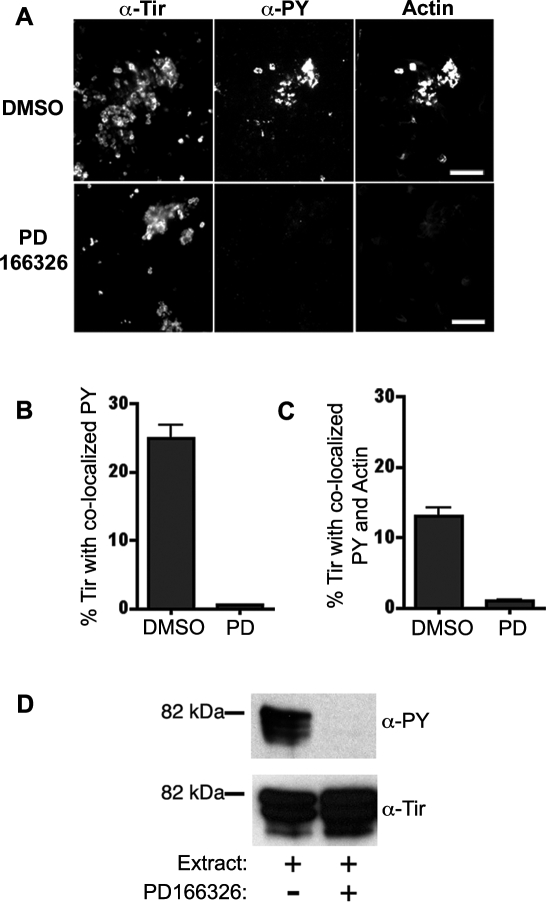
PD-166326 Blocks Actin Polymerization and Tir Phosphorylation in RBC Infected with EPEC and Exposed to Extract (A) Images of RBC infected with EPEC and exposed to low-ATP extract containing either 10 μM PD-166326 or an equivalent dilution of carrier (DMSO). Cells were stained with anti-Tir antibody, anti-PY 4G10 antibody, and Alexa-488-phalloidin to visualize actin. (B) Quantitative analysis of colocalization of PY staining and Tir in RBC infected with EPEC and exposed to low-ATP extract containing either 10 μM PD-166326 or DMSO. (C) Quantitative analysis of colocalization of actin staining with PY and Tir in RBC infected with EPEC and exposed to low-ATP extract containing either 10 μM PD-166326 or DMSO. (D) Western analysis using anti-PY 4G10 antibody and anti-Tir antibody of the TX-100 soluble fraction of RBC infected with EPEC and exposed to low-ATP extract containing 10 μM PD-166326.

Previous studies have indicated that Abl is sufficient for actin pedestal formation in intact cells, though other related kinases, such as Etk, also appear sufficient [10,11]. To determine whether Abl or related kinases participate in formation of pedestals in RBC treated with extract, we incubated infected RBC with extract that had been pretreated with PD-166326, a pyrido[2,3-d]-pyrimidine compound which blocks Abl- and Etk-family kinases by competing with ATP for binding in the SH1 domain of the kinase. PD-166326 has previously been shown to inhibit Tir phosphorylation and pedestal formation in EPEC infected 3T3 cells [11]. As a control, extracts were incubated with an equivalent concentration of DMSO, the solvent for PD-166326. Because PD-166326 and ATP compete, we formulated extracts without additional ATP and prepared an ATP regeneration mix with the minimal concentration of ATP required to support actin polymerization. As seen in Figure 5A, PD-166326 efficiently blocked actin polymerization and phosphotyrosine staining beneath attached bacteria, though it did not affect Tir localization. Quantitation of colocalization indicated that pretreatment of extract with PD-166326 significantly decreased phosphotyrosine staining and actin polymerization. (Figure 5B and 5C). Western analysis of the TX-100 soluble fraction derived from cells exposed to PD-treated extract confirmed that the compound completely blocked extract-induced tyrosine phosphorylation of Tir (Figure 5D). These data suggest that kinases present in the extract and subject to inhibition by PD-166326 mediate phosphorylation of Tir and actin polymerization induced by extract, as they do in intact cells [10,11].

Together, the data presented support the use of RBC infected with EPEC and treated with extract to define molecular mechanisms by which the pathogen induces actin polymerization. Such an extract system is particularly useful for dissecting events associated with infection that have not been recognized to depend on host factors in intact cells, or are not easily amenable to manipulation. We next use this system to identify a role for cytoplasmic factors in releasing Tir from EPEC and inserting it into the host plasma membrane, two of the earliest events in EPEC pathogenesis. We also define a role for cytoplasmic factors in facilitating phosphorylation of Tir by tyrosine kinases such as Abl.

### Extract Facilitates Release of Tir from EPEC and Translocation Into the Plasma Membrane

Although infection of RBC with EPEC results in large numbers of EPEC adhering to RBC, Shaw et al. [19] have reported that only a small proportion of attached EPEC appear to insert Tir into the RBC membrane under standard infection conditions. It is possible that RBC lack signaling components that efficiently activate secretion or translocation of Tir, or that such components washout out during hemolysis. In this regard, our analysis indicated that upon addition of extract, the quantity of Tir associated with the TX-100 soluble fraction of infected cells dramatically increases (Figure 4A). We reasoned that components in the extract might facilitate secretion of Tir by EPEC or insertion of Tir into the RBC membrane, or both.

**Figure 6:**
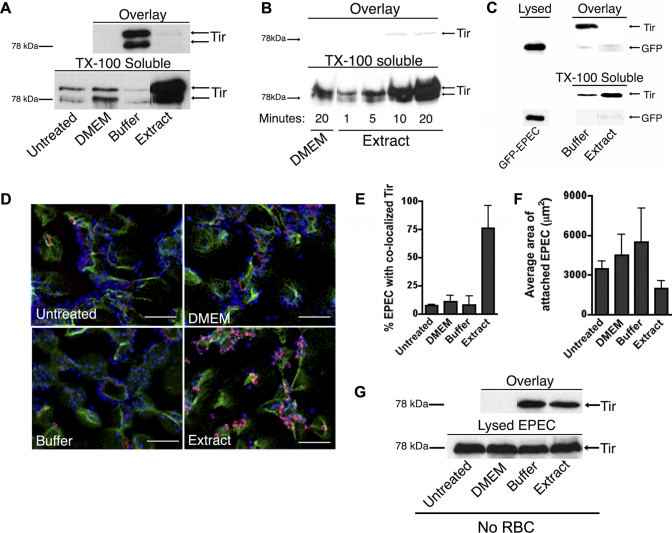
Extract Facilitates Release of Tir from EPEC and Translocation Into the RBC Membrane (A) Western analysis of RBC infected with EPEC and exposed to either DMEM, buffer, or extract for 20 min or left untreated and lysed immediately after infection. Both the Overlay and TX-100 soluble fractions were probed with anti-Tir antibody. (B) Western analysis of RBC infected with EPEC and exposed to either DMEM for 20 min (lane 1), or to extract for 1, 5, 10, or 20 min (lanes 2–5). Both the Overlay and TX-100 soluble fractions were probed with anti-Tir antibody. (C) Western analysis of RBC infected with GFP-EPEC and exposed to either buffer or extract for 20 min. Samples were probed with anti-GFP antibody to assess bacterial lysis and then stripped and reprobed with anti-Tir antibody. GFP-EPEC lysed in SDS-PAGE sample buffer and probed with anti-GFP antibody were run as a control to verify GFP expression (lane 1). Note that very little GFP is detected in either the overlay or the TX-100 soluble fraction after exposure to either buffer or extract. (D) Images of RBC infected with EPEC and exposed to either DMEM, buffer, or extract for 20 min or left untreated and fixed immediately after infection. Cells were stained with DAPI (blue) to visualize bacteria, anti-Tir antibody (red) and Alexa-488-phalloidin to visualize actin (green). Scale bar in all images represents 5 μm. Note the increase in Tir staining beneath EPEC attached to RBC after exposure to extract. (E) Quantitative analysis of colocalization of EPEC (DAPI) and Tir staining in RBC infected with EPEC and exposed to either DMEM, buffer, or extract for 20 min, or left untreated. (F) Quantitative analysis of colocalization of EPEC (DAPI) and RBC (visualized by Alexa-488-phalloidin) to assess the quantity of EPEC attached to RBC immediately following infection (untreated) or after exposure to either DMEM, buffer, or extract for 20 min. (G) Western analysis using anti-Tir antibody of EPEC cultured in DMEM for 6 h in the absence of RBC and then incubated with either DMEM, buffer, or extract for 20 min, or left untreated and lysed immediately. An aliquot of the supernatant was collected to assess Tir secretion (Overlay) and the bacterial pellet was lysed with SDS-PAGE sample buffer to assess bacterial Tir. Note that both buffer and extract induce secretion of Tir into the solution overlying the bacteria when no RBCs are present.

To investigate these possibilities, RBC monolayers were infected with EPEC, as in previous experiments, and either left untreated, a measure of the baseline level of Tir in the RBC membrane immediately following infection, or treated with DMEM, buffer or extract for 20 min. Following incubation, samples were assessed for both insertion of Tir into the RBC membrane (Figure 6A, TX-100 soluble) as well as secretion of Tir into the solution overlying the cells (Figure 6A, Overlay). Western analysis and densitometry revealed that treatment of the RBC monolayer with DMEM resulted in less than a two-fold increase in the amount of Tir in the TX-100 soluble fraction compared to untreated RBC. By contrast, RBC treated with extract contained as much as eight fold more Tir in the TX-100 soluble fraction compared to that seen in untreated RBC, or RBC incubated with DMEM, and 20 fold more Tir than RBC treated with buffer (Figure 6A). Notably, no Tir was detected in the solution overlying infected RBC upon treatment with DMEM or extract. However, after treatment of infected RBC with buffer alone, large amounts of Tir were evident in the solution overlying the cells with no increase evident in the TX-100 soluble fraction compared to untreated RBC. Analysis of RBC infected with EPEC and exposed to extract for 1 to 20 min indicated that the increase in Tir in the TX-100-soluble fraction became evident within 5 min, and that no Tir was released into the solution overlying the cells at any time point (Figure 6B). Thus, following infection in DMEM, continued exposure to this media induced little increase in Tir in the TX-100 soluble membrane fraction, and no secretion of Tir into the overlying solution. By contrast, exposure of infected RBC to buffer caused secretion of Tir exclusively into the solution overlying the cells, whereas exposure to extract induced rapid translocation and insertion of Tir into the RBC membrane.

To confirm that the increases in Tir seen in the TX-100 soluble fraction or in the overlying solution were not due to contamination from bacterial lysis, RBC were infected with EPEC expressing GFP (GFP-EPEC) and exposed to DMEM, buffer or extract. Both the TX-100 soluble and overlay fractions were analyzed by western analysis with anti-Tir antibody and anti-GFP antibody. The samples were not subjected to incubation at 90 °C to enrich for Tir to avoid precipitation of GFP from the samples. Although the increases in Tir detected in the various fractions following exposure to either buffer or extract were similar to those seen following infection with EPEC, GFP was only detected in the lysed bacteria control, indicating that little or no bacterial lysis had occurred under any condition (Figure 6C).

The increase in Tir seen in the TX-100 soluble fraction of infected RBC exposed to extract was also evident by fluorescence microscopy (Figure 6D). Quantitation of colocalization indicated a 5–7-fold increase in levels of Tir beneath attached bacteria upon incubation with extract compared to untreated RBC or RBC incubated with DMEM or buffer (Figure 6E). Notably, the increased levels of Tir in the TX-100 soluble fraction following treatment with extract could not be attributed to an increase in the number of bacteria attached to the cells as microscopy routinely revealed that fewer bacteria remained attached to RBC after treatment with extract as compared to treatment with DMEM or buffer (Figure 6F).

Previous reports suggest that growth of EPEC in DMEM in the absence of host cells results in secretion of components of the Type III secretion apparatus and expression, but not secretion, of Tir [30]. Accordingly, we found high levels of Tir upon lysis of EPEC cultured in DMEM for 6 h in the absence of RBC (Figure 6G; Untreated). Exposure of 6 h cultures of EPEC grown in the absence of RBC to either buffer or extract for 20 min prior to lysis caused no significant change in the amount of Tir within the bacteria; however, addition of either buffer or extract to 6 h cultures of EPEC induced rapid secretion of Tir into the overlying solution (Figure 6G). Taken together, these data suggest that exposure of EPEC to buffer or to extract induces rapid secretion of Tir from an existing pool within the bacteria, in the presence or absence of RBC. However, when EPEC infect RBC, components in extract appear to facilitate localization of secreted Tir to the RBC membrane.

### Decreased Ca^++^ Induces Rapid Release of Tir from EPEC

The observation that buffer or extract could initiate rapid secretion of Tir into the overlying solution, or the membrane, respectively, and the observation that no secretion was observed in DMEM, suggested that one or more of the components present in both extract and buffer might be responsible for inducing secretion of Tir from the bacteria under these conditions. In this regard, previous work has suggested that Ca^++^ concentration might regulate secretion of Type III effectors such as Tir. Thus, growth of EPEC in high calcium media such as DMEM (∼2 mM Ca^++^) stimulates secretion of Type III accessory translocation proteins EspA, B and D [31], whereas growth in low Ca^++^ media reduces secretion of proteins required for translocation and facilitates secretion of effector proteins such as Tir and NleA [30,32]. Notably, the homogenization buffer used in the preparation of extracts, and in the buffer controls, contained high concentrations of Ca^++^ chelators and both extract and buffer solutions were estimated to contain significantly lower free Ca^++^ concentrations (< 1 μM) than DMEM.

**Figure 7:**
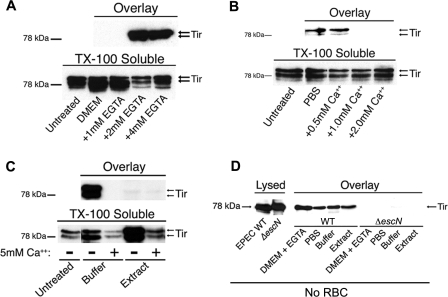
Decreased Ca^++^ Induces Rapid Release of Tir from EPEC (A) Western analysis of RBC infected with EPEC and exposed to either normal DMEM (2 mM Ca^++^) or DMEM supplemented with 1 to 4 mM EGTA. Both the Overlay and TX-100 soluble fractions were probed with anti-Tir antibody. (B) Western analysis of RBC infected with EPEC and exposed to either Ca^++^-free PBS or Ca^++^-free PBS supplemented with 0.5 to 2 mM CaCl_2_. Both the Overlay and TX-100 soluble fractions were probed with anti-Tir antibody. (C) Western analysis of RBC infected with EPEC and either left untreated, or exposed to buffer or extract with or without the addition of 5 mM CaCl_2_. Both the Overlay and TX-100 soluble fractions were probed with anti-Tir antibody. (D) Western analysis of wild-type EPEC (WT) and EPECΔ*escN* cultured in DMEM for 6 h in the absence of RBC and either lysed with SDS-PAGE sample buffer to verify Tir expression (lanes 1 and 2), or exposed to either DMEM + 4 mM EGTA, Ca^++^ free PBS, buffer or extract to induce Tir secretion (WT; lanes 3–6, *ΔescN*; lanes 7–10). Both the lysed bacteria and the Overlay fraction were probed with anti-Tir antibody. Note that although EPEC*ΔescN* contained large amounts of Tir when lysed, no secreted Tir was evident following exposure to low Ca^++^ conditions.

To test the possibility that a low Ca^++^ environment could induce rapid secretion of Tir from EPEC that had been cultured in DMEM, RBC were infected with EPEC, washed, and then incubated for 20 min with either normal DMEM, DMEM supplemented with increasing concentrations of the calcium chelator EGTA, or with Ca^++^-free PBS supplemented with increasing concentrations of free calcium. As shown in Figure 7A, only a small increase in the amount of Tir was evident in the TX-100 soluble fraction, and there was no secretion into the overlying solution, when the RBC monolayer was incubated in normal DMEM. However, addition of DMEM containing 2 to 4 mM EGTA dramatically increased the secretion of Tir into the overlying solution (Figure 7A). By contrast, secreted Tir was evident in the overlying solution after addition of Ca^++^-free PBS, but secretion was abolished at Ca^++^ concentrations above 1 mM (Figure 7B). Notably, no significant increase in Tir was evident in the TX-100 soluble fraction under any condition (Figure 7A and 7B), indicating that induction of Tir secretion by low Ca^++^ did not result in Tir translocation into the RBC membrane. Finally, treatment of infected RBC with either buffer or extract supplemented with excess Ca^++^ (5 mM) to contain the same free Ca^++^ concentration as DMEM, caused no secretion of Tir into the overlying solution and no significant increase in the amount of Tir in the TX-100 soluble fraction, similar to results seen with untreated RBC (Figure 7C). In accordance with previous reports [30,32], these data indicate that a low Ca^++^ environment can facilitate release of Tir from EPEC and demonstrate that Tir secretion is rapidly induced when cultured EPEC are switched from a high to a low Ca^++^ environment.

To confirm that Tir released from EPEC in low Ca^++^ was dependent on the Type III secretion system, we assessed secretion of Tir from EPECΔ*escN*. *EscN* encodes a putative ATPase of the Type III secretion apparatus and has previously been shown to be essential for Tir secretion into host cells and for pedestal formation [33]. We were not able to assess the effects of EPECΔ*escN* on RBC, because this mutant fails to adhere to or hemolyze RBC [34]. Instead, bacteria were cultured in DMEM and either lysed in SDS-PAGE sample buffer, or exposed to DMEM + 4 mM EGTA, Ca^++^-free PBS, buffer or extract, all of which contain low concentrations of free Ca^++^. Although all of the solutions induced secretion of Tir from EPEC, EPEC*ΔescN* failed to secrete Tir under any condition (Figure 7D). Tir was evident, however, upon lysis of EPEC*ΔescN* (Figure 7, lane 2), indicating that these bacteria did produce Tir. These data indicate that the rapid Tir secretion that occurs following transfer of EPEC from growth in DMEM to a low Ca^++^ environment requires a functional Type III secretion system.

### Heat-Labile Extract Components Facilitate Translocation and Anchoring of Tir in the Plasma Membrane

**Figure 8:**
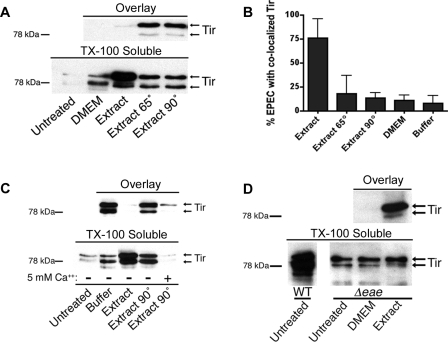
A Heat-Labile Cytosolic Factor(s) and Intimin Facilitate Translocation of Tir into the RBC Membrane (A) Western analysis of RBC infected with EPEC and exposed to either DMEM, extract, or heat-treated extract that was incubated at (65 °C for 15 min (Extract 65°) or incubated at 90 °C for 5 min (Extract 90°)), or left untreated and lysed immediately after infection. Both the Overlay and TX-100 soluble fractions were probed with anti-Tir antibody. (B) Quantitative analysis of colocalization of EPEC (DAPI) and Tir staining after exposure to either DMEM, buffer, extract, or heat-treated extract for 20 min. (C) Western analysis of RBC infected with EPEC and exposed to either buffer, extract, heat-treated extract, heat-treated extract plus 5 mM CaCl_2_, or left untreated and lysed immediately after infection. Both the Overlay and TX-100 soluble fractions were probed with anti-Tir antibody. (D) Western analysis of RBC infected with EPEC or EPECΔ*eae* and treated with either DMEM or extract for 20 min, or left untreated and lysed immediately after infection. Both the Overlay and TX-100 soluble fractions were probed with anti-Tir antibody.

The observation that exposure of infected RBC to low Ca^++^ solutions, such as buffer or PBS, induces an increase in Tir in the overlying solution, but not the TX-100 soluble fraction, suggests that insertion of Tir into the RBC membrane following exposure to extract, or anchoring of inserted Tir, occurs by a mechanism that does not depend on Ca^++^. We reasoned that factors from both the extract and the bacteria might contribute to insertion, anchoring or both. To begin to characterize factors within the extract other than calcium, extract was heated to 65 °C for 15 min, or 90 °C for five min, and centrifuged to remove precipitated material prior to incubation with RBC. Heat-treated extract never induced the dramatic increase in Tir in the TX-100 soluble fraction seen following exposure to untreated extract (Figure 8A). Moreover, exposure of infected RBC to heat-treated extract significantly increased the levels of Tir in the overlying solution (Figure 8A), a phenomenon not seen with untreated extract. Microscopy of infected RBC exposed to heat-treated extract also revealed no significant increase in Tir localization beneath attached EPEC compared to treatment with DMEM (Figure 8B). Addition of 5 mM Ca^++^ to heat-treated extract (resulting in ∼2 mM free Ca^++^) reduced secretion of Tir into the overlying solution and significantly reduced levels of Tir in the TX-100 soluble fraction (Figure 8C). Taken together, these data indicate that the low Ca^++^ concentration in extract induces secretion of Tir, but that a heat-labile factor(s) in the extract facilitates efficient insertion or anchoring of Tir in the RBC membrane.

The observed increase in TX-100 soluble Tir following treatment with extract was independent of Tir phosphorylation, as it was unaffected by pretreatment of extracts with PD-166326 (see Figure 5C). Likewise, removal of the ATP-regeneration system from extract also caused little change in levels of TX-100 soluble Tir (Figure S1C). Thus, insertion or anchoring of Tir within the RBC membrane does not require ATP at levels necessary to support Tir phosphorylation or actin polymerization. Finally, the increased levels of Tir in the TX-100 soluble fractions of RBC treated with extract were not simply the result of the overall increased protein concentration of extract compared to buffer. Thus, addition of BSA to buffer at a concentration comparable to the total protein concentration of extract produced only a modest change in Tir in both the TX-100 soluble and secreted fractions compared to treatment with extract or buffer lacking BSA (Figure S1D).

### Intimin Facilitates Translocation or Stabilization of Tir in the RBC Membrane

The possibility existed that bacterial components might also facilitate translocation of Tir into the RBC membrane, or its stabilization once inserted. Intimin has been shown to bind to Tir and to play a vital role in clustering Tir in the host cell membrane [4,35]. To determine if intimin was also necessary for the increase in membrane associated Tir seen after the exposure to extact, RBC were infected with an EPEC mutant lacking intimin (EPECΔ*eae*) and exposed to either DMEM, buffer or extract or left untreated. As seen in Figure 8D, baseline levels of Tir were lower in the TX-100 soluble fractions of RBC infected with EPECΔ*eae* compared to wild- type EPEC (lanes 1 and 2) and RBC monolayers infected with EPECΔ*eae* showed no increase in levels of TX-100 soluble Tir following exposure to extract. Instead, in contrast to wild-type EPEC, exposure to extract induced secretion of Tir into the overlying solution. These data suggest that bacterial intimin is likely not involved in the initiation of Tir secretion following exposure to extract, but is required for the insertion or stabilization of Tir in the RBC membrane following secretion.

### Cytoplasmic Factors Facilitate Phosphorylation of Tir by Tyrosine Kinases

**Figure 9:**
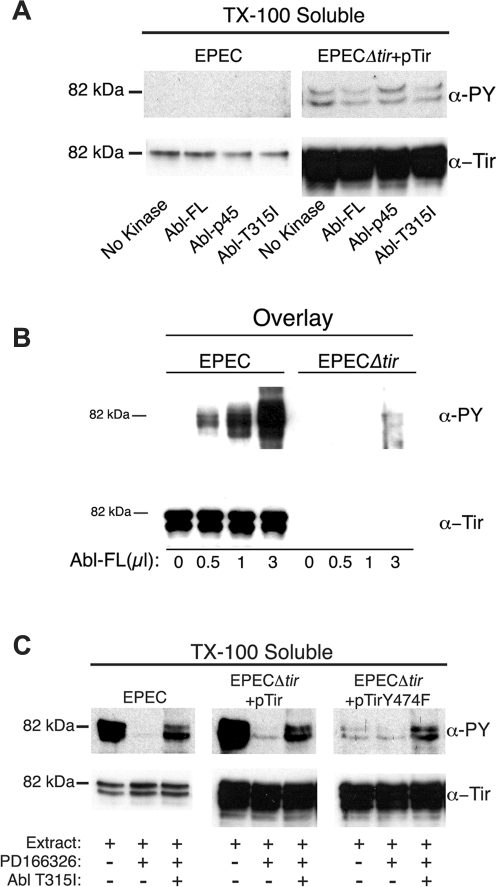
Tyrosine Kinases Complexed with Cellular Factors Are Required for Phosphorylation of Tir (A) Western analysis of RBC infected with EPEC, or EPEC*Δtir*+pTir, and exposed to DMEM (supplemented with ATP and TX-100) with or without the addition of purified Abl-FL, Abl-p45, or Abl-T315I kinase (2 μl) for 20 min at 37 °C. Samples were blotted and probed with anti-PY 4G10 antibody and then stripped and reprobed with anti-Tir antibody. (B) Western analysis of RBC infected with either EPEC-WT or EPECΔ*tir* and exposed to buffer containing increasing quantities of purified Abl-FL kinase (0–3 μl) for 20 min at 37 °C. The solution overlying the cells (Overlay) was collected to assess phosphorylation of secreted Tir and samples were blotted as in (A). (C) Western analysis of TX-100 soluble fraction of RBC infected either with EPEC, EPEC*Δtir*+pTir, or EPEC*Δtir*+pTiry474F and incubated with extract alone, with extract supplemented with 10 μl PD-166326, or additionally with 2 μl Abl-T315I for 20 min at 37 °C. Samples were blotted as in (A).

Previous reports indicate that both Abl and Src are capable of phosphorylating Tir isolated by immunoprecipitation from intact cells, and that the Abl- and Etk-family kinases are sufficient for Tir phosphorylation in intact cells [10] We next determined whether Abl alone was sufficient to phosphorylate Tir localized in the RBC plasma membrane. To do this, purified active full-length Abl kinase lacking only the first N-terminal 27 amino acid residues (Abl-FL), or truncated active form containing only the SH2 and catalytic domains (Abl-p45), was added to DMEM supplemented with ATP and TX-100, and incubated with infected RBC. Kinase assays were conducted in tandem in the same buffer to assess the capacity of each kinase to phosphorylate GST-Crk, a known Abl substrate. As seen in Figure 9A (left panel), western analysis indicated that no tyrosine phosphorylation of Tir was evident in the TX-100 soluble fractions of samples following addition of either Abl-FL or Abl-p45 at 2 μl per reaction, even when the blot was exposed to film overnight. However, as little as 0.2 μl μlof either kinase readily phosphorylated GST-Crk under the same reaction conditions, indicating that the kinases were active (data not shown). To determine if the lack of Tir phosphorylation following exposure to purified Abl was simply due to low levels of Tir found in the RBC membrane following exposure to DMEM, RBC were infected with EPEC*Δtir+*pTir, and incubated with DMEM plus Abl-FL or Abl-p45 (Figure 9A, right panel). Infection with EPEC*Δtir+*pTir results in high levels of Tir in the TX-100 soluble fraction of infected cells compared to infections with wild-type EPEC. Nevertheless, no Tir phosphorylation could be detected following exposure to DMEM plus the purified kinases compared to DMEM alone. When purified Abl-FL was added to buffer, instead of DMEM, and layered over infected RBC, thus inducing secretion of large amounts of Tir into the overlying solution, phosphorylation of Tir was readily evident in the overlay fraction following addition of as little as 0.5 μl of kinase (Figure 9B). No phosphorylation was evident under these conditions when RBC were infected with EPEC*Δtir*. Together these data suggest that although purified Abl can readily phosphorylate Tir that has been secreted into the overlying solution, phosphorylation sites on Tir within the RBC membrane are not readily accessible to exogenous kinase under these conditions.

Because extract readily phosphorylates Tir in the RBC membrane, we next tested whether cofactors present within the extract facilitate access of kinases to Tir. To do this, we assessed whether exogenous Abl, added together with extract, could support phosphorylation of Tir in infected RBC. To isolate the effects of exogenous Abl from those kinases within the extract that phosphorylate Tir and support actin polymerization, extract kinases were inhibited by addition of PD-166326 (see Figure 5), and Abl-T315I, a variant of Abl that is resistant to inhibition by PD-166326 [11] was added instead of Abl-FL. RBC were infected with either EPEC, EPEC*Δtir*+pTir or EPEC*Δtir*+pTirY474F and exposed to either extract alone, PD-166326-treated extract or PD-166326-treated extract + Abl-T315I. As shown in Figure 9C (left and middle panel), phosphorylation of Tir in the PD-166326-treated extract was evident upon addition of Abl-T315I in RBC infected with both EPEC and EPEC*Δtir*+pTir, though to a lesser extent than that seen upon addition of extract alone. However, the phosphorylation did not appear to be specific for Y474 because the same level of Tir phosphorylation was evident in RBC infected with EPEC*Δtir*+pTirY474F (Figure 9C, right panel). As with DMEM plus Abl-FL or Abl-p45, no phosphorylation of Tir was detectable in the TX-100-soluble fraction upon addition of DMEM together with Abl-T315I in the absence of extract, even when Tir was over-expressed (Figure 9A). Together these data indicate that phosphorylation of Tir by tyrosine kinases in infected RBC requires cofactors in the extract that are likely complexed with endogenous kinases. We hypothesize that these cofactors are present in such limiting amounts as to be unavailable for use by exogenously added kinases.

## Discussion

### Reconstituting Cytoskeletal Functions with Extracts

The recapitulation of cellular functions using cell-free extracts has led to seminal discoveries in cell biology, particularly relating to the regulation of cytoskeletal function. Moreover, the coupling of extract systems with microbial pathogens has proven immensely useful in understanding the components regulating actin polymerization. Theriot and Mitchison recapitulated actin-dependent cytoplasmic motility of Listeria monocytogenes in cell-free extracts [21] and, using actin polymerization induced by *Listeria* as a read-out, Welch and Mitchison fractionated extracts and identified the components of the Arp2/3 complex [23]. Efforts by Kirschner and others have been directed at defining the cellular regulators of the Arp2/3 complex using protein complexes purified from cell-free extracts [6,36]. More recently, *Listeria* motility in extracts has been used as a means to study the dynamics and mechanics of actin polymerization [37].

A limitation of cell free systems has been the inability to identify membrane components that regulate actin polymerization from the cell surface. Permeabilized cells supplemented with monomeric actin have been useful for identifying sites of new polymerization [26]. However, the trauma induced by permeabilization has proven a significant limitation in the reconstitution of signaling that mediates actin polymerization induced by extracellular signals. Indeed, efforts to study actin polymerization on membranes have proven most successful in the context of vesicular movement [20].

In an effort to address this limitation, we describe here an extract-based RBC system that recapitulates the rapid formation of actin pedestals induced by EPEC in the plasma membrane of intact cells. Such a system is unique in that it allows dissection of the delivery mechanisms for virulence factors into the host cell membrane as well as the mechanisms of actin polymerization and membrane protrusion occurring at the plasma membrane. Moreover, cytoplasmic regulators of these processes can be identified by biochemical fractionation methods and manipulated experimentally in a time frame that is physiologically relevant. Pedestal formation and actin polymerization in extract-treated RBC induced by EPEC utilize host proteins such as Nck, N-WASP, and the Arp2/3 complex, which regulate actin polymerization in response to a wide variety of stimuli [14,15,20,38–40]. Thus, we anticipate that the system may prove useful in dissecting signaling by transmembrane receptors to actin.

Previous efforts to study actin polymerization induced by Nck and N-WASP in extracts were catalyzed by a 12-mer phosphopeptide from EPEC Tir containing residues surrounding Y474 [6]. However, several lines of evidence suggest that the Tir phosphopeptide may not be a physiologically relevant stimulus. First, for N-WASP activation to occur *in vitro*, other components, such as PIP_2,_ must also be added [36,38], raising the possibility that, like tyrosine kinases, N-WASP activation requires multiple stimuli. Second, results from our lab [10] suggest that the Tir phosphopeptide alone cannot initiate activation of N-WASP and formation of actin pedestals; pedestal formation instead requires stable tyrosine kinase recruitment via polyproline residues and residues including and surrounding phosphorylated Y474 within Tir interacting with SH3 domains and SH2 domains within tyrosine kinases, respectively. The extract system described here may allow understanding of the complexes regulating actin polymerization at cellular membranes.

### Does Actin Polymerization Induced by EPEC in RBC Resemble Pedestals on Intact Cells?

The dependence of actin polymerization on PD-166326-sensitive kinases and signaling via Nck, N-WASP, and Arp2/3 complex all point to a mechanistic similarity between the two systems. However, unlike intact cells, we found little evidence for elongated actin structures in the RBC system. We speculate that several factors may account for such a difference. First, it is possible that key bacterial virulence factors that contribute to elongation “wash out” in the permeabilization process, or that the extract lacks factors that facilitate elongation. In this regard, Kenny and colleagues have noted a role for serine and threonine modifications of Tir in elongation [8]. Likewise, Rosenshine and colleagues have described a virulence factor called EspH from EPEC and EHEC that contributes to pedestal elongation [41]. Our results (Figure 4) and those of Kenny using exogenous Tir in permeabilized cells [42] indicate incomplete molecular weight shifts compared to Tir translocated into intact cells via type III secretion. Second, the membrane of RBC may be less susceptible to protrusive forces either because it has higher rigidity than other cellular membranes due to differences in composition [43], or because anchoring sites for actin are unavailable. Finally, it is possible that the intrinsic depolymerizing or capping activity in the extract is higher than in cytoplasm of intact cells. Nevertheless, the capacity to recruit signaling proteins to the membrane remains intact, and identification of novel recruited proteins will likely provide important information on the regulation of complexes of proteins that initiate actin polymerization at the membrane.

### Using the RBC-Extract System to Define Host Factors That Mediate Type III Secretion

How do host proteins facilitate virulence factor translocation via the Type III secretion system? Previous reports have defined mutants in a wide variety of bacterial proteins that are involved in either the assembly of the Type III secretion apparatus or the secretion of virulence factors once bacteria have attached to a host cell (e.g., EspA, EspB, and EspD in EPEC and their homologues in other pathogens [44]). Moreover, various factors, including calcium, have been suggested to control secretion of effector proteins by both *Yersinia* and EPEC based on measuring secretion of virulence factors into culture supernatants in the absence of host cells [30–32,45]. However, a role for host factors in triggering secretion of virulence factors into host cells or facilitating their transport and targeting within the host cell has been difficult to assess. The RBC system described here has allowed us to determine that cellular factors such as calcium indeed participate in the rapid release of Tir via the Type III secretion system, and additionally, that a heat-labile host cell factor and bacterial intimin are both required to facilitate insertion and anchoring of Tir into the host cell plasma membrane following secretion.

The effects of calcium on Type III secretion have been noted previously in several bacterial cell types [30,32,45,46]but these studies have usually assessed protein secretion into culture media following several hours of incubation and have been carried out in the absence of host cells. Our data suggest that Tir secretion and translocation into the RBC membrane is initiated within 5 min following exposure to low calcium and persists for as long as 20 min, the longest time tested. Accordingly, Wolff et al. have reported that secretion of EspB from EPEC occurs within ten min following host cell contact [47], and time-lapse microscopy of fluorescently labeled effector proteins secreted by *Shigella* has indicated that half of the bacterial pool of each of these proteins is secreted from individual bacterium within 2–6 min of stimulation with Congo Red [48]. Our data provide support for the view that the Type III secretion apparatus has a calcium sensor that can distinguish the micromolar concentration inside a host cell from the millimolar level outside the cell and can thus determine when the secretion needle has achieved access into the host cytoplasm, thus signaling the bacterium to induce rapid secretion of virulence factors into the host cell. However, we cannot rule out the possibility that calcium may mimic the physiological signal for Tir secretion rather than being the signal itself.

The RBC system we have developed may be ideally suited for investigating how host factors participate in Type III secretion because, as noted in a previous report [19] only limited amounts of Tir are inserted into the RBC plasma membrane during the normal course of infection. We presume that cytoplasmic factors within RBC are limiting, or that, following hemolysis, such factors wash out. Addition of extract, however, results in a rapid increase in Tir in the membrane, which can be detected by western analysis and fluorescence microscopy. In previous studies [42,49] there has been controversy as to whether Tir in the TX-100 soluble fraction comprises only Tir in the plasma membrane or whether contamination from bacterial cell lysis occurs. However, our data with GFP-EPEC suggest that Tir from lysed bacteria represents a relatively small fraction of the Tir in the TX-100 soluble fraction. Moreover, Tir levels assessed by microscopy are in accordance with those seen biochemically.

Shaw et al. demonstrated with immuno-EM and immunofluorescence microscopy that Tir integration and colocalization with intimin occur with RBC in the absence of added cytoplasmic extract. So the question arises as to how Shaw and colleagues were able to visualize Tir-intimin interactions even without added cytoplasm. Notably, Shaw et al. used EPEC that had been serially passaged over several days, or coinfected RBC with EPECΔ*espA* together with EPEC*Δtir*. Under both these conditions, an enhancement of Tir and intimin expression was evident. By contrast, out experiments utilize wild-type EPEC from an overnight culture. So, the effect of cytosol on membrane levels of Tir is evident because we have not used conditions that boost Tir expression. In accordance with the results of Shaw et al., no effect of cytosol is evident when infecting with an EPEC strain that markedly over-expresses Tir. Thus, our data suggest that extract facilitates efficient insertion of Tir into the plasma membrane.

Our data suggest that Tir secreted from bacteria upon low calcium stimulation or upon addition of heat-treated extract does not enter the membrane efficiently. Indeed, under these conditions, large amounts of Tir are detected in the overlay solution with only small changes in the TX-100 soluble fraction compared to untreated samples. However, previous reports indicate that Tir added to the outside of cells can enter the plasma membranes directly [50], though it remains to be resolved whether Tir is transported into the cytoplasm en route to the membrane. We envision two possible scenarios by which cytoplasmic factors increase levels of Tir in the plasma membrane. In the first, a cytoplasmic factor analogous to a bacterial chaperone inserts Tir into the plasma membrane. We speculate that a PH domain-containing protein that uses PIP_3_ as membrane dock may act in this capacity. Second, a cytoplasmic factor may alter the membrane thereby facilitating insertion of Tir. Resolution of this issue awaits a more precise understanding of the mechanisms by which host factors insert and stabilize Tir in membrane. We are currently fractionating the extract as a means to define both the host factor or factors and the mechanism by which Tir is inserted into the plasma membrane. We anticipate that such a factor may be utilized by many pathogens that translocate virulence factors into host membranes.

### Using the RBC-Extract System to Define Host Factors That Mediate Tyrosine Phosphorylation of Tir

Data presented here suggest that cytoplasmic factors also participate in phosphorylation of Tir by tyrosine kinases. Although Tir in solution appears to be susceptible to phosphorylation by exogenous tyrosine kinases such as Abl and Src [10], our experiments suggest that in permeabilized RBC, only endogenous kinases in the extract that are sensitive to PD-166326 are capable of phosphorylating Tir on Y474 and supporting actin polymerization. We hypothesize that cofactors in the extract are necessary to facilitate functional phosphorylation of Tir on Y474 and that these cofactors are only present in limited quantities and are complexed with endogenous kinases. Together, these data suggest that tyrosine kinases within the extract are within complexes that regulate both their activity and their capacity to recognize substrates in the membrane.

In infected RBC, Tir phosphorylation can still occur under conditions that preclude actin polymerization beneath bacteria (e.g., in solutions lacking TX-100), and the polymerization machinery does not appear to be recruited in the presence of tyrosine kinase inhibitors. Thus, neither actin nor the polymerization machinery appears to be necessary to target tyrosine kinases to Tir. In this regard, evidence from intact cells suggests that phosphorylation of Tir by kinases such as Abl1, Abl2, or Etk may induce a positive feedback loop in which initial phosphorylation of Tir begets recruitment of kinases, which in turn phosphorylate Tir and other cellular factors required for pedestal formation [10]. Additionally, tyrosine kinases such as Src and Abl contain myristoyl groups that allow targeting to the plasma membrane, and which are required for activity [51]. Data presented here suggests that for Abl recruitment to occur, the kinase must be assembled into a complex. Current efforts are directed at purifying the components of this complex.

### Summary

The extract system described here provides a means to purify and identify cytoplasmic components that facilitate insertion and anchoring of Tir in the plasma membrane as well as the formation of actin pedestals. Moreover, the system permits manipulation of cytoplasmic signaling factors through extract depletion and supplementation, or the use of chemical inhibitors, and provides a means to reconstitute pedestal formation using purified components to help further characterize the dynamics of signaling events associated with EPEC virulence factor translocation, Tir phosphorylation and actin polymerization at the plasma membrane.

## Materials and Methods

### Bacterial strains, antibodies, and reagents.

The EPEC strain E2348/69 was used for infections. EPECΔ*tir,* EPECΔ*eae* and EPECΔ*escN* strains were provided by J. Kaper (University of Maryland), and the EPECΔ*tir* + pTir and EPECΔ*tir* + pTirY474F were provided by B. Bommarius (Emory University; [10]). The antibodies used were as follows: anti-Tir (a gift from J. Kaper, University of Maryland) was used at 1:10,000 for microscopy and 1:50,000 for westerns, anti-phosphotyrosine (PY) 4G10 (Upstate) was used at 1:250 for microscopy and 1:1,000 for westerns, anti-WASP that also recognizes N-WASP (a gift from Jack Taunton, USCF), anti-Arp2/3 (a gift from Matthew Welch, UC Berkeley), and anti-Nck (BD Biosciences Pharmingen) were used at 1:100 for microscopy, anti-GFP antibody (BD Biosciences) was used at 1:5,000. For microscopy, bacteria were visualized with DAPI (5 mg/ml; Roche) and actin with phalloidin conjugated to either FITC or Alexa-488 (6 mU/ml; Molecular Probes) and secondary antibodies conjugated with various fluorophores (Jackson Immunochemicals) were used at 1 mg/ml. The following purified kinases were used: Abl-p45 (NEB, 100,000 U/ml), Abl-FL (Upstate Biotechnologies, 1351U/mg) and Abl(T315I) (Upstate Biotechnologies, 605U/mg). The tyrosine kinase inhibitor PD-166326 was provided by W. Bornmann (M.D. Anderson Cancer Center) and was used at 10 μM.

### Preparation of brain cytosolic extract.

Cytosolic extract was prepared from either freshly harvested whole brain from C57BL/6 mice (Jackson Laboratories) or from frozen unstripped porcine brain (Pel-Freez). Brain tissue was suspended in ice cold Homogenization Buffer (20 mM Hepes pH. 7.2, 50 mM KCl, 2 mM MgCl_2_, 5 mM EGTA, 1 mM EDTA, and 100 mM sucrose with protease inhibitors (Roche Complete) and 0.5 mM ATP (added just before use) at a ratio of 0.75 ml buffer per gram of wet weight, and immediately homogenized on ice using a teflon dounce homogenizer attached to an electric drill (Makita Co.). After homogenization, DTT was added to a final concentration of 0.5 mM and samples were centrifuged at 26,000 × g for 20 min. The resulting supernatant was harvested and subjected to a second high-speed spin of 104,330 × g for 1 h. The supernatant was snap frozen and stored in aliquots at −80 °C. Because occasional variation between batches of extract could not be avoided, each batch of extract was tested for the ability to induce actin polymerization beneath EPEC on infected RBC prior to use in experiments. After testing extracts prepared from a variety of fresh and frozen tissue sources, we found that those prepared from frozen porcine brain (PelFreez) yielded the most consistent results and these were used for the majority of the experiments presented. The data presented in Figures 1 and 2 was obtained using murine brain extract. Repeating these experiments with porcine extract yielded identical results. The data presented in the remaining figures was obtained using porcine extract. Extracts stored at −80 °C for more than 6 mo were found to yield inconsistent actin polymerization and were discarded.

### RBC plating and infection.

Human red blood cells were isolated from whole blood by dextran sedimentation [52]. RBC plating and infection were carried out as described by Shaw et al. [16,19] with minor modifications. Briefly, sedimented RBC were washed with sterile PBS and pelleted at 600 × g at 4 °C (3 times). RBC were plated as a 3% solution in PBS on PDL/Collagen coated glass cover slips for microscopy, or 24 well tissue culture dishes for western analysis, and allowed to settle for 20 min. Wells were washed with PBS to remove unattached RBC until only a single layer of cells could be observed adhering directly to the plating surface. EPEC was cultured overnight in Luria broth at 37 °C immediately prior to infections. Infections of RBC monolayers were carried out in sodium bicarbonate buffered Dulbecco's Modified Eagles Medium (DMEM) in a 37 °C, 5% CO_2_ incubator at a ratio of 10 μl of overnight EPEC culture per ml of media. Infection times were 6–8 h. Shaw et al. found that infection of RBC with wild-type EPEC from a typical overnight culture resulted in only minimal numbers of EPEC translocating Tir and showing Tir/intimin intimate attachment. Infection with serially passaged EPEC (4 d) was necessary to enhance the level of Tir translocation and intimate attachment to detectable levels. In our experiments, infection of RBC with serially passaged EPEC did not result in any significant increase in actin polymerization compared to infection with a typical overnight (data not shown). We therefore did not employ this method and used only single EPEC overnight cultures for all experiments presented. Indeed, this procedure allowed us to visualize increases in Tir upon addition of extract.

### Cytosolic extract reactions.

Cytosolic extract reaction mixture was a modification of that used by Theriot et al. [21] to generate Listeria actin tail formation in cell-free extracts and was composed of three separate solutions that were mixed immediately prior to use: (i) ATP regeneration solution (150 mM creatine phosphate, 20 mM ATP, 2 mM EGTA pH 7.7, 20 mM MgCl_2_); (ii) Reaction solution (0.14 mg/ml creatine kinase, 0.14% TX-100); and (iii) Brain Cytosolic Extract (prepared as described above) . The final mixture (which is referred to in the text as “extract”) was combined in a ratio of 1 μl ATP Regenration mix, 13 μl Reaction Solution, and 20 μl Brain Cytosolic Extract in a final volume that was determined independently for each experiment depending on number of samples and volume needed per sample. The final concentrations for all components was as follows: 4 mM creatine phosphate, 0.6 mM ATP, 0.05 μg/μl creatine kinase, 0.053% TX-100, 11.7 mM Hepes, 29 mM KCl, 1.6 mM MgCl_2_, 2.96 mM EGTA pH 7.7, 0.6 mM EDTA, 59 mM sucrose. For buffer controls, the Brain Cytosolic Extract was replaced with Homogenization Buffer (see Preparation of Brain Cytosolic Extract above), but the rest of the components remained the same (this mixture is referred to in the text as “buffer”). After infection, RBC were washed with PBS to remove unattached EPEC. Untreated samples were processed immediately after infection without any exposure to buffer or extract. Buffer and Extract treated samples were layered with either buffer or extract at a volume sufficient to completely cover the RBC monolayer. All samples were incubated at 37 °C for 20 min unless otherwise noted. After incubation, the solution overlaying the cells was collected (Overlay) and the RBC monolayer was washed with PBS and one or both fractions were processed for western analysis or immunofluorescence microscopy as described below. For experiments with EPEC in the absence of RBC, bacteria were grown in DMEM for 6–8 h and equivalent numbers of bacteria were placed in tubes and exposed to the various conditions for 20 min. Samples were centrifuged to pellet bacteria between experimental solutions and washes.

### Kinase assays.

To assess the ability of purified Abl kinase to phosphorylate Tir in the RBC membrane in the absence of extract, infected monolayers were washed with PBS and layered with either buffer (formulated as above for cytosolic extract reactions) or DMEM (plus 0.6 mM ATP and 0.05% TX-100) supplemented with 0.5–2 μl of the various purified Abl kinases (Abl-FL, Abl-p45, Abl-T315I). Samples were incubated at 37 °C for 20 min, washed with PBS and processed for western analysis or microscopy as described below. To verify kinase activity in DMEM, 0.2 μl of kinase was added to DMEM containing 0.6 mM ATP, 0.05% TX and 1 μg of Abl kinase substrate GST-Crk and incubated at 37 °C for 20 min. To assess Tir phosporylation by purified Abl in the presence of extract, infected monolayers were washed with PBS and layered with extract that had been pretreated with 10 μM PD-166326 for 5 min prior to addition of 2 μl of purified Abl. Samples were incubated at 37 °C for 20 min, washed with PBS and processed for western analysis or microscopy as described below. Addition of kinases was not based on units because kinase activities were not directly compared between kinases and kinase unit definitions differed by manufacturer.

### Cell fractionation and western analysis.

To asses membrane-associated Tir (TX-100 soluble fraction), washed RBC monolayers were lysed on ice with Triton X-100 Lysis buffer [42] (20 mM Tris pH 8.0, 150 mM NaCl, 5mM EDTA, 1% TX-100, 10% glycerol, protease inhibitor mix (Roche Complete) and phosphatase inhibitor mix (Calbiochem) for 20 min, scraped from the plate and centrifuged (13,000 × g for 5 min) to remove insoluble material and whole EPEC. To assess secreted Tir that did not become associated with the TX-100 soluble fraction, an aliquot of the reaction solution overlaying the cells was collected prior to washing the monolayer and centrifuged to remove contaminating bacteria (“Overlay”). Untreated samples do not have a corresponding overlay fraction. In most experiments we have applied a method established by Kenny and colleagues, in which supernatants from both the TX-100 soluble and Overlay fractions were heated to 90 °C for 5 min and subjected to a second centrifugation to remove precipitated material and enrich for Tir in the supernatant [29]. Unfortunately, this procedure destroys cellular proteins that could be used as loading controls (e.g., tubulin and actin). To normalize samples we have used a procedure adapted from immunoprecipitation experiments. In particular, we have started with equivalent amounts of cellular material and loaded equivalent volumes of supernatant containing Tir. Normalization using protein assays was complicated by addition of bacteria or extract and the heating step. In many experiments, duplicate wells were used and gave the identical results. In the figures, we have removed duplicate lanes from gels for ease of presentation, but all blots and controls were from the same experiment, and all experiments were repeated at least three times. Final supernatants were subjected to SDS-PAGE and western blotting according to standard protocols. Blots probed with more than one antibody were stripped (Re-Blot Plus, Chemicon) of the first antibody and verified to have no residual signal prior to analysis with the second antibody. ImageJ densitometry software (NIH) was used to quantitate relative changes in Tir following western analysis.

### Immunofluorescence staining and microscopy.

Detailed methods for fixation and immunofluorescent staining have been previously described [10,11,15]. Images were acquired with a scientific grade cooled charge-coupled device (Cool-Snap HQ) on a multi-wavelength wide-field microscopy system (Intelligent Imaging Innovations) based on a Zeiss 200M Axiovert microscope using either a 63X N.A. 1.4 lens or 40X N.A. 1.3 lens (Zeiss). Immunofluorescent samples were imaged at room temperature using a standard Sedat filter set (Chroma) in successive 0.2 mm focal planes through the z-plane of the sample. Out-of-focus light was removed with a constrained iterative deconvolution algorithm [53]. For each condition or experiment, image data was collected on the same day using identical exposure times.

### Quantitation of colocalization.

To quantitate the degree of colocalization of fluorescence signals from several fluorophores, we first defined the area of fluorescence and the fluorescence intensity within that area for each channel in control and experimental images (12 images per condition captured randomly on the slip with a 40x N.A.1.3 lens). The acquisition parameters used for all images under all conditions were identical on a given day. Prior to analysis, the contrast and intensity settings were adjusted to equivalent levels for all channels in images. The area of fluorescence for each channel was chosen using the MASK function in the Intelligent Imaging Innovations software package. Briefly, we generated a precise “map” of the location of fluorescent objects of interest (e.g., bacteria) apparent in the image based on intensity. To do this, we first analyzed each channel of a control image and adjusted the threshold of the function so that objects of greater or lesser intensity than the object in question were excluded. For example, within the DAPI channel we chose intensities corresponding to the bacteria but excluded those corresponding to background staining, which were of lower relative intensity. We then applied these parameters for a given channel to all channels within all control and experimental images in the data set to generate a set of masks. So each mask for each channel contained a spatial representation (i.e., coordinates and areas) of pixels within an intensity range that corresponded to objects of interest (i.e., bacteria, actin, and Tir staining). Within each image, we then combined masks of different channels with a boolian AND function to define a new mask representing the area of colocalization. We then calculated the area of overlap. For example, we calculated the area of overlap of Tir and PY or Tir and actin. We normalized the value to the total area of Tir signal within each image. The average and standard error values were then calculated based on data from twelve images. Each experiment was repeated at least three times. Representative images and data from a single experiment are displayed in the Figures.

## Supporting Information

Figure S1 Further Analysis of Extract-Induced Secretion, Membrane Insertion, and Phosphorylation of Tir (A) Western analysis of Tir in the TX-100 soluble fraction of RBC infected with either EPEC or EPEC*Δtir* and exposed to extract for 20 min. (B) Western analysis of Tir in the TX-100 soluble fraction of RBC exposed to extract or buffer for 5, 15, or 30 min. Infected RBC were exposed to extract for 5, 15, or 30 min with (lanes 2, 4, and 6) or without (lanes 1, 3, and 5) phosphatase inhibitor (Na_3_VO_4_) Note that by 30 min, Tir phosphorylation is significantly diminished, even in the presence of phosphatase inhibitor. (C) Western analysis of RBC infected with EPEC and exposed to either DMEM, extract or extract without the components of the ATP regeneration system (−ATP). Samples were probed with anti-Tir antibody. (D) Western analysis of RBC infected with EPEC and exposed to buffer, extract or buffer plus 12 mg/ml BSA (equivalent to average total protein concentration of extract). Samples were probed with anti-Tir antibody.
(380 KB PDF)

Figure S2 Larger Representation of Figure 4D Images of RBC infected with EPEC*Δtir* mutants over-expressing either WT Tir (*Δtir* + pTir ) or Y474F Tir (*Δtir* + pTirY474F) and exposed to extract for 20 min. Cells are labeled with DAPI to identify EPEC, Alexa-488-phalloidin to visualize actin and either anti-Tir antibody or anti-PY 4G10 antibody. Note the lack of actin polymerization and PY staining in RBC infected with EPEC*Δtir* + pTirY474F. Scale bars represent 10 μm.
(680 KB PDF)
