# Supplementary material for: Cytosolic Extract Induces Tir Translocation and Pedestals in EPEC-Infected Red Blood Cells
Source: PLoS Pathog. 2008 Jan 18;4(1):e4. doi: 10.1371/journal.ppat.0040004 (PMC2211550; doi:10.1371/journal.ppat.0040004)
Supplement: Supplementary file 1 [file ppat.0040004.sg001.pdf]

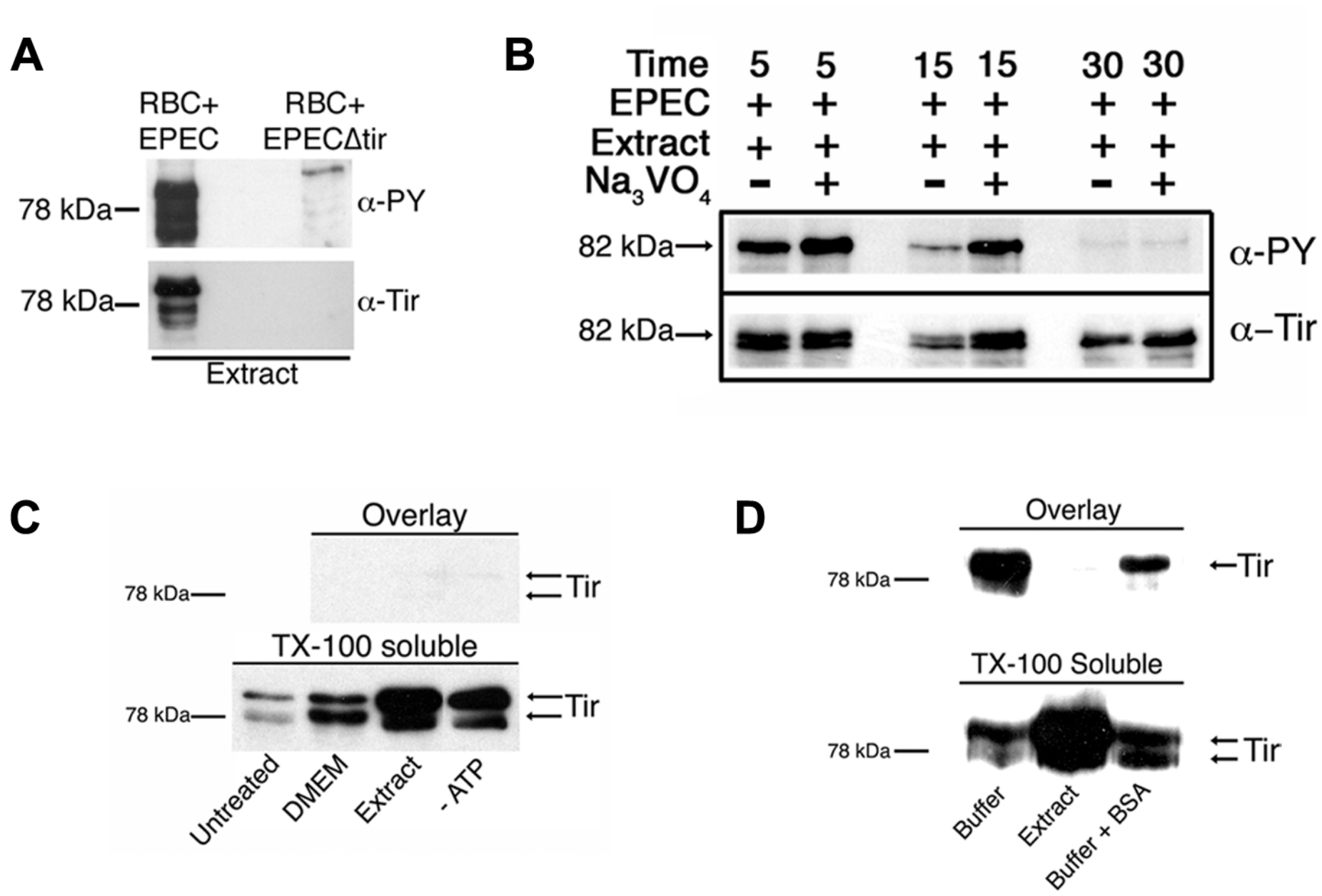
A
RBC+ EPEC
RBC+ EPECΔtir
78 kDa
α-PY
78 kDa
α-Tir
Extract
B
Time 5 5 15 15 30 30
EPEC + + + + + +
Extract + + + + + +
Na3VO4 - + - + - +
82 kDa
α-PY
82 kDa
α-Tir
C
Overlay
78 kDa
Tir
TX-100 soluble
78 kDa
Tir
Untreated
DMEM
Extract
- ATP
D
Overlay
78 kDa
Tir
TX-100 Soluble
78 kDa
Tir
Buffer
Extract
Buffer + BSA
