# Supplementary material for: Cytosolic Extract Induces Tir Translocation and Pedestals in EPEC-Infected Red Blood Cells
Source: PLoS Pathog. 2008 Jan 18;4(1):e4. doi: 10.1371/journal.ppat.0040004 (PMC2211550; doi:10.1371/journal.ppat.0040004)
Supplement: Supplementary file 2 [file ppat.0040004.sg002.pdf]

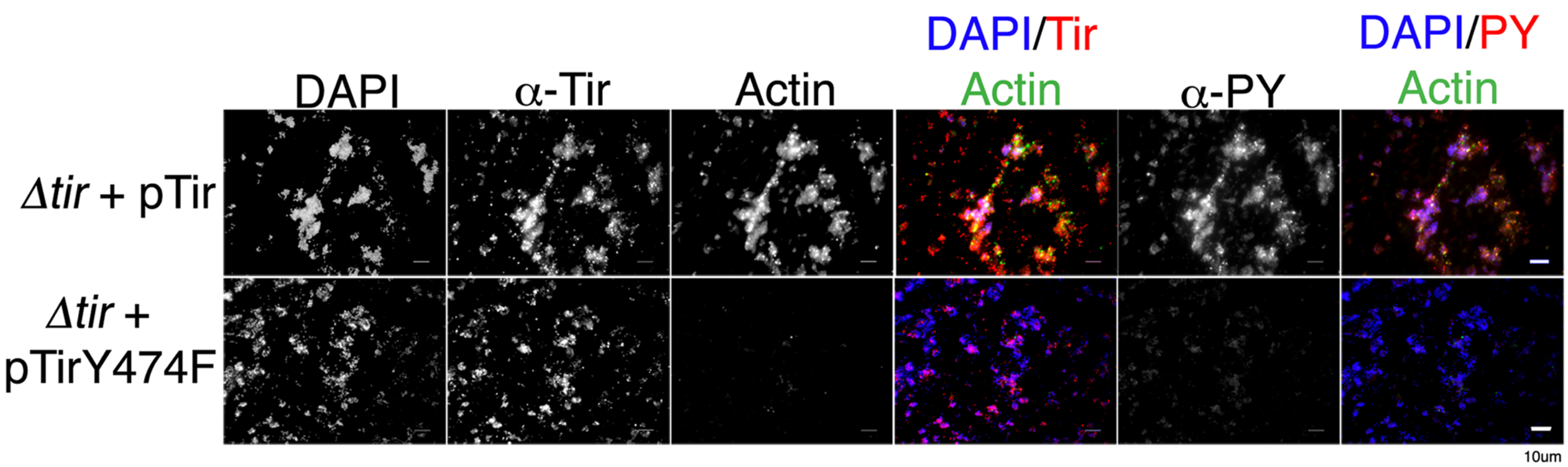

DAPI
α-Tir
Actin
DAPI/Tir
Actin
α-PY
DAPI/PY
Actin
Δtir + pTir
Δtir +
pTirY474F
10um
